# A Cognitive Behavioral Therapy–, Biofeedback-, and Game-Based eHealth Intervention to Treat Anxiety in Children and Young People With Long-Term Physical Conditions (Starship Rescue): Co-design and Open Trial

**DOI:** 10.2196/26084

**Published:** 2021-09-24

**Authors:** Hiran Thabrew, Karolina Stasiak, Harshali Kumar, Tarique Naseem, Christopher Frampton, Sally Merry

**Affiliations:** 1 Department of Psychological Medicine University of Auckland Auckland New Zealand; 2 Carbon Imagineering Auckland New Zealand; 3 Statistical Consultants Limited Auckland New Zealand

**Keywords:** long-term physical conditions, chronic illness, anxiety, eHealth, gaming, young people, treatment, cognitive behavioral therapy, biofeedback

## Abstract

**Background:**

Approximately 10%-12% of New Zealand children and young people have long-term physical conditions (chronic illnesses) and are more likely to develop psychological problems, particularly anxiety and depression. Delayed treatment leads to worse health care and poorer long-term outcomes. Recently, eHealth interventions, especially those based on principles of cognitive behavioral therapy and biofeedback, have been shown to be moderately effective in reducing anxiety. However, these modalities have rarely been combined. Young people have expressed a preference for well-designed and technology-based support to deal with psychological issues.

**Objective:**

This study aims to co-design and evaluate the acceptability and usability of a cognitive behavioral therapy and biofeedback-based, 5-module eHealth game called *Starship Rescue* and to provide preliminary evidence regarding its effectiveness in addressing anxiety and quality of life in young people with long-term physical conditions.

**Methods:**

*Starship Rescue* was co-designed with 15 children and young people from a tertiary hospital in New Zealand. Following this, 24 others aged 10-17 years participated in an open trial of the game, accessing it over an 8-week period. The acceptability of the game to all participants was assessed using a brief, open-ended questionnaire. More detailed feedback was obtained from a subset of 10 participants via semistructured interviews. Usability was evaluated via device-recorded frequency and duration of access on completion of the game and the System Usability Scale. Anxiety levels were measured at baseline, completion, and 3 months after completion of the game using the Generalized Anxiety Disorder 7-item scale and Spence Child Anxiety Scale, and at the start of each module and on completion using an embedded Likert visual analog scale. Quality of life was measured at baseline, completion, and 3 months after completion using the Pediatric Quality of Life Inventory scale.

**Results:**

Users gave *Starship Rescue* an overall rating of 5.9 out of 10 (range 3-10) and a mean score of 71 out of 100 (SD 11.7; minimum 47.5; maximum 90) on the System Usability Scale. The mean period for the use of the game was just over 11 weeks (78.8 days, 13.5 hours, 40 minutes). Significant reductions in anxiety were noted between the start and end of the game on the Generalized Anxiety Disorder 7-item scale (−4.6; *P*<.001), Spence Child Anxiety Scale (−9.6; *P*=.005), and the Likert visual analog scales (−2.4; *P*=.001). Quality of life also improved on the Pediatric Quality of Life Inventory scale (+4.3; *P*=.04). All changes were sustained at the 3-month follow-up.

**Conclusions:**

This study provides preliminary evidence for *Starship Rescue* as an acceptable, usable, and effective eHealth intervention for treating anxiety in young people with long-term physical conditions. Further evaluation is planned via a randomized controlled trial.

**Trial Registration:**

Australian New Zealand Clinical Trials Network Registry (ANZCTR) ACTRN12616001253493; https://www.anzctr.org.au/Trial/Registration/TrialReview.aspx?id=371443

## Introduction

Long-term physical conditions (also known as chronic illnesses), defined as those lasting more than 3 months and impairing functioning, are common, affecting 10%-12% of children globally [1]. Such conditions include asthma, diabetes, epilepsy, and obesity, among others [2,3]. The prevalence of long-term physical conditions in childhood is increasing [4]. Owing to improvements in hygiene, immunization, and access to medical care in some high-income countries, it is greater than that of acute illness [5].

Psychological problems are more likely in children and young people with long-term physical conditions [6-11]. Of these, anxiety is the most common, with some studies identifying rates as high as 40% [12]. The likelihood of psychological problems, including anxiety, appears to be related to numerous factors that may impose a cumulative allostatic load [13]. These include developmentally related self-regulation, family dynamics, illness, and procedure-related pain or distress [13,14] and readjustment to normal life following the completion of treatment [15]. In the longer term, untreated anxiety may have a chronic and unremitting course [16] and increase the risk of other psychiatric problems, such as depression and substance use disorders [17].

Access to and effectiveness of treatments for psychological problems in children and young people with long-term physical conditions are currently limited. Although they are traditionally addressed using generic psychotherapies, such as cognitive behavioral therapy (CBT) and pharmacotherapies such as anxiolytic or antidepressant medication, there is limited evidence that such therapies are effective for this population [18]. Even in the general population, CBT only has a 60% response rate for anxiety treatment, suggesting room for improvement [19]. In addition, access to psychotherapies is often limited and dependent on the availability of community child and adolescent mental health services, pediatric consultation-liaison services, and other health services. Most interventions designed for children and young people with long-term physical conditions focus on adherence to medical treatment, education about medical conditions, and improving aspects of medical care [18].

Over the past few decades, the increasing popularity of smart technology, release of gamified and app-based interventions, and calls from international organizations, such as The Lancet Global Mental Health Group [20] for the introduction of innovative and accessible cognitive and behavioral strategies to treat anxiety, depression, and other common mental health problems, have led to the likelihood that eHealth interventions will play a significant role in future mental health delivery. Purported advantages of eHealth interventions include increased accessibility, greater anonymity, flexibility, reduced expenses, eliminated travel time, and interactivity [21,22]. Several recent systematic reviews have confirmed the effectiveness of eHealth interventions for anxiety in young people, the most recent of these citing moderate to large effect sizes compared with no treatment (*g*=0.53-1.41) [23-27]. The most widely used and evaluated eHealth interventions for childhood anxiety are the CBT-based interventions BRAVE (Body Signs, Relaxation, Active Helpful Thoughts, Victory Over Your Fears, Enjoy) online [28] and the Cool Kids series that includes Little Cool Kids for younger children [29], Cool Kids online for older children [30], and Chilled Out for adolescents (the latter was developed from a CD-ROM version called Cool Teens) [31]. Both have been shown to be clinically effective, but none address anxiety in the context of health-related conditions, nor are they widely available outside Australia. A number of other CBT-based interventions with evidence of effectiveness also exist [32]. A few mindfulness-based interventions, such as Personal Investigator and an unnamed problem-solving intervention, also have limited evidence of acceptability and user satisfaction [32]. To date, no eHealth interventions have been specifically designed to address anxiety in children and young people with long-term physical conditions. Given the medically related and unrelated factors that lead to anxiety in this group and the fact that anxiety management needs to be available and effective in the context of ongoing physical health care, it seems likely that they have different needs from the general population. A recent Cochrane review identified only one CBT-based, chronic pain–focused, web-based intervention, Web-MAP. Furthermore, 2 low-quality studies provided unclear evidence of their effectiveness in reducing anxiety [33].

Traditional psychological therapies often include a component of psychologically or chemically induced relaxation. There is increasing evidence that newer, more technology-based forms of therapy, such as biofeedback, may achieve similar results, either alone or in combination with traditional therapies [34]. Furthermore, some biofeedback interventions have already been combined with game-based technology to reduce stress or treat behavioral disorders [35]. Biofeedback involves the use of electrical or electromechanical equipment to measure physiologic processes occurring in a person and then feed this information back to them to develop a greater awareness and ability to control changes within their bodies with and without equipment [36] and improve health and performance [37]. There are several types of biofeedback, including heart rate variability (HRV), electroencephalography, and pneumography. Two HRV biofeedback-based interventions, Dojo and Relax to Win, have been demonstrated to reduce childhood anxiety [32]. A third electroencephalography, mindfulness, and CBT-based intervention—Mindlight—has also shown some promise [32]. A recent systematic review supported HRV as the most effective form of biofeedback for the treatment of anxiety and supported further research into hybrid models of therapy [38].

In a recent study, New Zealand young people with long-term physical conditions confirmed that anxiety is the most significant psychological issue that they face [39]. Together with their families and clinicians, they described limited knowledge of and access to eHealth interventions and expressed support for the development of eHealth interventions targeted toward their needs. Between 2017 and 2019, a 6-month co-design process was undertaken with 15 New Zealand young people and a local game developer (Carbon Imagineering) to develop a CBT and biofeedback-based eHealth game called *Starship Rescue* [40]. This involved 3 cycles or *sprints* during which the prototype game was developed and *scrums* during which feedback was collected and reviewed. At the end of each cycle, a deliverable version was made available to the tester group to garner further feedback, which was then implemented in the next development cycle. Following this, an open trial was undertaken with the aim of evaluating its (1) acceptability and (2) usability and (3) ability to provide preliminary evidence of its clinical effectiveness before conducting a randomized controlled trial (RCT).

## Methods

### Design

The open trial, conceptualized by 3 authors (HT, KS, and SM), used a mixed-methods design, including quantitative analysis of anxiety symptoms and quality of life outcomes, intervention use, and qualitative analysis of participant feedback.

### Population

A total of 24 young people aged between 10 and 17 years were recruited from a tertiary children’s hospital in Auckland, New Zealand, between October 2018 and May 2020. Eligible participants had any type of long-term physical condition lasting for longer than 3 months (eg, asthma, diabetes, cancer, and cystic fibrosis) and measurable levels of anxiety (eg, specific phobia, generalized anxiety, and nonspecific anxiety) and may or may not have had comorbid mental health conditions. Eligible participants were of any ethnicity, intellectually and physically able to use the intervention, and understood enough English to play the game and provide informed consent or assent with paired parental consent if they were aged <16 years. Participants who did not meet all these criteria and those who had recently undertaken or were undertaking CBT or other forms of psychotherapy, biofeedback therapy, or pharmacotherapy with anxiolytic medication within the past 6 months were excluded because the effect of those therapies could confound the impact of the intervention.

### Intervention

**Figure 1:**
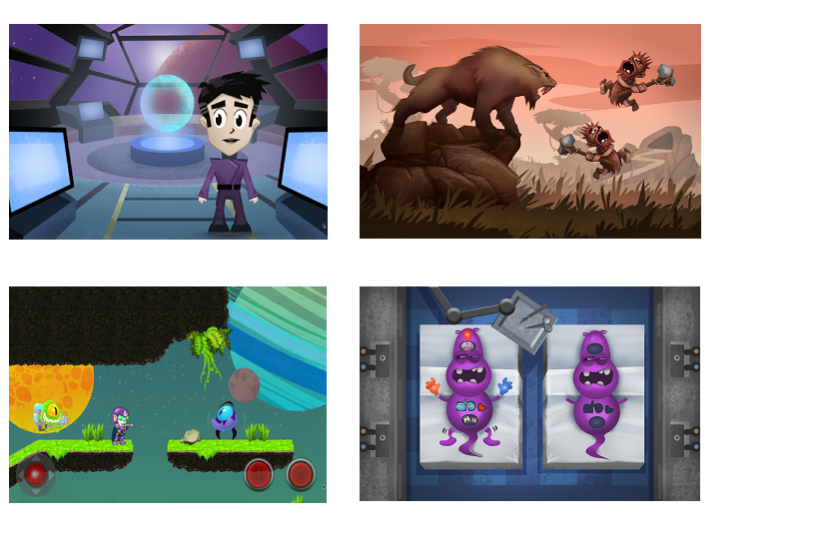
Illustrative images from the game—clockwise from top left: bridge of starship; learning about the origins of anxiety; exploring the anxiety monster; planet of the mind.

*Starship Rescue* is a game-based eHealth intervention based on the story of a space hospital (starship) that gets caught up in a vortex of anxiety. The narrative is a new captain whose mission is to help find the lost bravery stars and restart its engine. Purposely designed to harness the correlation between shorter duration of use and outcomes [41], it includes 5 modules, each taking between 15 and 30 minutes to complete. Module 1 introduces players to anxiety and its origins and features, module 2 focuses on beating anxiety using their bodies, for example, via deep breathing and progressive muscle relaxation, module 3 teaches players how to discern between helpful and unhelpful thoughts and to prioritize the former, module 4 introduces problem solving and graded exposure to address smaller and bigger forms of worry or anxiety, and module 5 is a final quiz to consolidate the learning from previous modules. On completion of the game, players are emailed a summary of learned techniques in the form of a stay cool capsule. The intervention is provided on a tablet synced with a commercially available, wrist-based Scosche Rhythm Plus heart rate monitor which is accessed during biofeedback-based relaxation exercises. Starship Rescue is underpinned by the principles of (1) CBT, (2) biofeedback, (3) learning theory, and (4) game player taxonomy. Although most knowledge and skills are gained within modules, users must also leave the game and practice overcoming a named worry or anxiety in the real world between the fourth and fifth modules to complete the intervention. No external therapist support is required. Parental involvement has previously been shown to aid the successful completion of eHealth interventions, learning, and application of skills and systemic risk factors associated with the maintenance of childhood anxiety [42]. During the use of Starship Rescue, parents help their child choose a real-life reward to receive on completion of the intervention, validate the achievement of an out-of-game task in the fourth module by entering a four-digit code so that they can proceed to the final module, and are emailed a summary of the key learning points if their child does not have an email address. Participants were loaned a tablet with Starship Rescue installed on it and encouraged to complete all modules at home or in the hospital within 8 weeks. If they requested additional time at this stage, it was provided. Player data were saved within the game, allowing participants to pause and resume the game when participants wished to do so. Some data (eg, module completion and time taken) were manually pushed by a member of the research team from the game to an administrator email address at the end of the game. Further details of the modules and theoretical underpinnings are provided in Multimedia Appendix 1 [43-54]. Illustrative images are presented in Figure 1.

### Outcome Measures

The primary outcomes of the open trial were evaluated as follows: (1) acceptability of the prototype intervention (ie, is the content and format acceptable to users?) was quantitatively assessed via user ratings of overall acceptability and helpfulness on scales from 0 to 10 and qualitatively assessed via feedback during semistructured interviews following completion of the game; (2) usability of the intervention (ie, is it usable?) was quantitatively assessed using the System Usability Scale (SUS) [55], time taken to complete the game and module completion and qualitatively assessed via feedback during semistructured interviews following completion of the game; and (3) clinical effectiveness (ie, does it reduce anxiety and related issues?) was assessed by measuring changes over time in the Generalized Anxiety Disorder, 7-item (GAD-7) [56], Spence Children’s Anxiety Scale (SCAS) [57], a Likert scale of anxiety embedded in the game, and the Pediatric Quality of Life Inventory (PedsQL) [58], as outlined in the schedule below (Table 1). The GAD-7 is a brief (7-item), self-reported scale for measuring anxiety in people aged ≥13 years. It has a sensitivity of 89% for generalized anxiety disorder, and scores of 5, 10, and 15 out of a possible 21 points indicate mild, moderate, and severe anxiety levels, respectively. The SCAS is a well-validated but longer (46-item) self-reported scale measuring child anxiety with sound psychometric properties with internal consistency reported at 0.92 for the total child score. It contains 6 subscales for panic or agoraphobia, social phobia, separation anxiety, obsessions or compulsions, fear of physical injury, and generalized anxiety. Likert scales (linear scales with items or numbers, eg, 0-10) have been shown to be useful for monitoring changes in anxiety [59] but are limited by user avoidance of extreme ratings [60]. Visual analog scales (involving images such as faces of differing sizes or nature) have also been shown to be useful for rating anxiety [61] with superior measurement qualities [62]. There is some disagreement about which type of scale is better for use with children [63,64]. Repeated brief evaluation of anxiety using simple measures such as Likert visual analog scales within a game, such as *Starship Rescue*, can be considered a form of ecological momentary assessment (EMA). EMA has been demonstrated to be useful for providing a richer picture of how behavioral changes occur over time [65]. To embed a repeated and accessible EMA within *Starship Rescue*, a combined Likert visual analog scale was developed that includes the face of an anxiety monster (as shown in Figure 1). This face can be moved with a slider to the desired point from the left (also marked 0) side of the screen to the right (also marked 10) and enlarges as this occurs. The PedsQL is a well-validated, 23-item self-report or parent-report scale measuring the quality of life. It has good internal consistency (0.88 for total scale), validity, and acceptability. It reliably distinguishes between healthy children and those with acute or long-term physical conditions.

**Table 1:** Schedule of outcome measurement.

Outcome	Start of game	Start of each module	Completion of game	3 months following completion
Acceptability	N/A^a^	N/A	User feedback via questionnaires and semistructured interviews	N/A
Usability	N/A	N/A	System Usability Scale, user feedback via semistructured interviews	N/A
Effectiveness	GAD-7^b^, SCAS^c^, Likert VAS^d^, PedsQL^e^	Likert VAS	GAD-7, SCAS, Likert VAS, Peds QL	GAD-7, SCAS, Peds QL

^a^N/A: not applicable.

^b^GAD-7: Generalized Anxiety Disorder-7 item.

^c^SCAS: Spence Children’s Anxiety Scale.

^d^VAS: visual analog scale.

^e^PedsQL: Pediatric Quality of Life Inventory.

### Statistical Methodology

Quantitative data were analyzed by our biostatistician (CF) using Excel (version 16, Microsoft Inc) and SPSS (version 25, IBM Corp). Analyses included basic descriptive statistics (eg, number of sessions completed, number of times device accessed, duration of use, changes in anxiety score, and demographic characteristics of the sample). McNemar chi-square tests and one-tailed *t* tests were used to assess the statistical significance of changes in anxiety scores over time. *P* values of <.05 were taken to indicate statistical significance, and 95% CIs were used to establish the extent of any difference between pre- and postmeasures. A sample size of at least 20 was calculated a priori to detect changes within the study group with effect sizes of 0.65 or more as statistically significant (α=.05 with 80% power). Data from this trial will be used to inform power calculations for a more definitive RCT. An intention-to-treat analysis was used with missing data managed using the last observation carried forward method. Qualitative data were manually analyzed using a general inductive approach [66] with collated text independently analyzed by 2 researchers (HT and HK) and any discrepancies addressed by consensus.

### Ethics and Consent

This study received ethics approval from the New Zealand Health and Disability Ethics Committee (HDEC, 16/CEN/136) on September 30, 2016. The lower age limit for participation was initially set at 12 years but later extended down to 10 years following a period of slow recruitment. Invitations to participate in the study were forwarded to potential participants through their own clinicians to minimize coercion using a direct approach. Verbal and written consent was obtained directly for those aged >16 years and via their parents with paired participant assent for those aged <16 years. Participants were free to discontinue engagement at any stage without consequence, and this was made clear to them. Although plans were made for any unanticipated distress occurring during participation to be managed by immediate referral to the hospital-based pediatric consultation-liaison mental health team, of which the lead author (HT) is a team member, this never occurred. Data were securely stored on a department server and kept securely for 10 years (or 10 years following younger participants’ 16th birthday) according to the ethics committee requirements.

## Results

### Feedback and Alterations to the Intervention From the Co-design Process

A total of 15 participants aged between 8 and 16 years, of mixed gender (10 males and 5 females) and with different long-term physical conditions (cancer, asthma, bronchiectasis, cystic fibrosis, Alport syndrome, and others) provided feedback, 2 of which on multiple occasions. User feedback was incorporated to address technical issues, make instructions clearer, and develop the game’s look and feel. Examples of user feedback during the first sprint and the use of this feedback are provided in Textbox 1. By the end of the third cycle, there were sufficiently minimal technical issues and common concerns to proceed with the open trial.

**Examples of feedback during the first sprint of co-design process.**

**Feedback and Proposed Alterations**

- Generally positive feedback regarding look or feel, for example, “It’s fun,” “I liked how some monsters chase you, and others need to be found.”
  None
- Technical issue identified: “Only one little bug, getting stuck in the block.”
  To be fixed by game developer
- Unsure whether different colored crystals are the same
  Clarification to be added to introduction to module 3
- Hard to recall positive and negative feelings when asked
  Summary list to be added to the end of module 1
- Re. ideal audience for the game: “I think younger kids, probably 8-15 years, any (boys and girls)”
  None, current game probably appropriate for target age range

### Open Trial

#### Participant Characteristics

A total of 32 participants (different from those who participated in the co-design process) were referred by their clinicians to participate in the open trial of the *Starship Rescue*. Of these, 24 met all the inclusion criteria and agreed to participate (Table 2). The most common long-term physical conditions were cancer (4/32, 12%), transplant (heart, liver, and kidney; 4/32, 12%), epilepsy (2/32, 6%), juvenile idiopathic arthritis (2/32, 6%), and nut allergy (2/32, 6%). Individual participants also had stroke and nonepileptic events (1/32, 3%); asthma (1/32, 3%); cystic fibrosis (1/32, 3%); nemaline rod myopathy and restrictive lung disease (1/32, 3%); cardiovascular disease, not specified (1/32, 3%); eczema (1/32, 3%); spina bifida (1/32, 3%); chronic fatigue syndrome and postural orthostatic tachycardia syndrome (1/32, 3%); long QT syndrome (1/32, 3%); type 1 diabetes; and celiac disease (1/32, 3%). A total of 3 participants did not wish to participate after the study was fully explained. Furthermore, 3 participants had inadequate anxiety symptoms (GAD-7 score <5). One participant denied having any anxiety at all, and 1 participant did not respond to multiple invitations.

**Table 2:** Participant characteristics (n=24).

Characteristics			Values
Age (years), mean (range)			14 (10-17)
**Sex, n (%)**			
	Male	9 (38)	
	Female	15 (63)	
**Long-term physical condition, n (%)**			
	Cancer	4 (17)	
	Transplant (heart, liver, and kidney)	4 (17)	
	Epilepsy	2 (8)	
	Juvenile idiopathic arthritis	2 (8)	
	Nut allergy	2 (8)	
	Other	10 (42)	

#### Acceptability

Participants gave *Starship Rescue* an overall rating of 5.9 out of 10 (SD 1.87; range 3-10) and a helpfulness rating of 6.3 out of 10 (SD 2.52; range 2-10). Qualitative feedback consisted of two main themes: helpfulness for managing anxiety and ease and enjoyment of use. The latter included 2 subthemes of positive and negative feedback, as presented with supporting examples in Table 3.

**Table 3:** Qualitative feedback regarding acceptability.

Theme and subtheme		Supporting examples (participant number)
Helpfulness for managing anxiety		“I enjoyed the games and thought the game gave quite good techniques.” [P13] “During the games where you had to keep your heart rate down, and breathing exercises, I did find ways to slow down my breathing, and calm my heart rate, which was good.” [P8] “It taught me a lot of breathing skills.” [P14] “The game points out very helpful things that you don’t really think about.” [P15]
**Ease and enjoyment of use**		
	**Positive feedback**	
		“The game was fairly easy to control and fairly smooth running.” [P5] “It was informative and the animations were fun.” [P4] “The heart rate monitor was fun - to see where my heart was at.” [P1] “It was really fun and I would do it again.” [P18]
	**Negative feedback**	
		“The game was too difficult in module 3.” [P4] “Bit too much talking and felt like module 2 was the same as module 1.” [P20] “I don’t feel like the game was for my age (15 years) and not enough shooting.” [P5]

#### Usability

Participants had mixed views on the usability of *Starship Rescue*. The game received an overall mean score of 71 out of 100 (SD 11.7; minimum 47.5; maximum 90) on the SUS. Almost two-thirds (13/24, 54%) of participants rated it above 68, defined by the scale’s authors as indicating *average usability*. More detailed SUS subscales are presented in Table 4. The module completion varied, as shown in Table 5. Despite the recommendation to use the game over a 4-week period, participants spent an average of 78.8 days (11-50 days; with one participant taking 243 days) to achieve completion. As we were keen for participants to complete the game during this pilot study to provide us with feedback to inform the design of a future RCT and as there was no way to retrieve the tablets until participants had finished playing the game, the duration of use varied considerably between participants and modules, as described in Table 5. Qualitative feedback regarding the game’s usability addressed technical issues, location of use, parental involvement, and recommendations for future improvement of the game. Further details are presented in Textbox 2.

**Table 4:** System Usability Scale subscales.

System Usability Scale item^a^	Values, mean (SD; range)
I thought the game was easy to use (+)	4.18 (0.92; 1-5)
I found the various functions in this game were well-integrated (+)	3.77 (1.05; 2-5)
I would imagine that most people would learn to use this game very quickly (+)	3.91 (0.87; 1-5)
I felt very confident using the game (+)	3.84 (1.20; 2-5)
I think that I would like to use this game frequently (+)	2.22 (1.02; 1-4)
I found the game unnecessarily complex (−)	1.91 (1.11; 1-5)
I found the game very cumbersome to use (−)	2.95 (0.67; 1-5)
I think that I would need support of a technical person to be able to use this game (−)	1.45 (0.81; 1-3)
I thought there was too much inconsistency in this game (−)	1.66 (1.20; 1-4)
I needed to learn a lot of things before I could get going with this game (−)	1.57 (0.90; 1-4)

^a^(+) higher scores indicate greater usability; (−) lower scores indicate decreased usability.

**Table 5:** Time taken to complete each module and the whole game.

	Completion (n=24), n (%)	Values, mean (SD; range)
Module 1	23 (96)	12.4 days (41.1; 11 minutes-142.9 days)
Module 2	23 (96)	5.6 days (7.2; 14 minutes-19.7 days)
Module 3	19 (79)	25.3 days (38.6; 26 minutes-128.3 days)
Module 4	17 (71)	13.6 days (18.7; 19 minutes-51.1 days)
Module 5	16 (67)	3.8 minutes (0.0006; 3.0 minutes-5.0 minutes)
Total	N/A^a^	79.4 days (9.52; 12.0 days-243.9 days)^b^

^a^N/A: not applicable.

^b^On the basis of participants with completed data for all five modules.

**Qualitative feedback regarding usability.**

**Technical Issues**

- “Some controls were a bit touchy and pressing the back button on the tablet would reset the progress on that module.” [P1]
- “Module three was difficult to pass.” [P11]
- “The games sometimes took a while to get the hang of.” [P3]

**Location of Use**

- “Just at home in my room.” [P19]
- “At home.” [P15]

**Parental Involvement**

- “Sometimes, if I didn’t know what to do...I asked my parents, or my bigger brother.” [P15]
- “[My mum] was actually quite involved; she just asked questions about it.” [P19]

**Recommendations for Improvement of the Game**

- “Make cut scenes skippable and add sections/chapters to each module.” [P8]
- “Add a pause button that automatically pauses the game if you leave, so you don’t lose progress.” [P15]
- “Disable the back button or use a different tablet.” [P1]
- “Have less backstory about the Starship and a more detailed description on how to play the mini-games.” [P13]
- “Add a double jump bar.” [for module 3; P17]

#### Effectiveness

Participants reported concordant changes in anxiety using three separate scales: GAD-7 [31], SCAS [32], and a Likert visual analog scale embedded in the game. The overall scores improved on all three scales with statistical significance (*P*<.005), as shown in Table 6. The overall effect size of the intervention was 0.6 (Cohen *d*). The change in anxiety using the Likert visual analog scale showed a positive association with the SCAS (*r*=0.59) and the GAD-7 (*r*=0.44). According to the GAD-7 scores, most participants (18/21, 86%) experienced a downgrading of symptom category (between severe, moderate, mild, and subthreshold) postintervention, whereas a few (3/21, 14%) remained the same. These changes were sustained at 3 months following completion, with the majority (15/21, 71%) continuing to report improvement, and some (6/21, 28%) remained the same (Multimedia Appendix 2). The numbers were too small to perform any reliable statistical calculations. Participants also reported improved quality of life using the PedsQL, as described in Table 6.

**Table 6:** Change in anxiety on General Anxiety Disorder-7 item, Spence Children’s Anxiety Scale, and Likert visual analog scales and quality of life on the Pediatric Quality of Life Inventory scale.

	Generalized Anxiety Disorder, 7-item scale			Spence Children’s Anxiety Scale				Likert visual analog scale			Pediatric Quality of Life Inventory scale	
	Pre	Post	3 months	Pre	Post	3 months	Pre		Post	Pre		Post
Participant, n	24	21	22	23	21	22	18		16	24		21
Value, mean (SD; range)	9.9 (5.4; 0-21)	5.3 (3.2; 1-12)	6.2 (4.7; 0-21)	35.7 (16.0; 13-69)	26.1 (13.9; 12-62)	26.0 (16.0; 8-79)	6.2 (1.5; 3.5-9.0)		3.8 (1.9; 0-7.5)	63.7 (15.9; 28.33-93.33)		68.0 (15.5; 40-88.33)
*P* value (vs prelevel)	N/A^a^	<.001	.001	N/A	<.001	.005	N/A		.001	N/A		.04

^a^N/A: not applicable.

## Discussion

### Principal Findings

Our findings provide preliminary evidence that *Starship Rescue* is an acceptable, usable, and effective new eHealth intervention for treating anxiety and improving quality of life in children and young people with long-term physical conditions. They also confirmed the feasibility of undertaking a larger RCT to confirm these findings. *Starship Rescue* appears to have comparable effectiveness (Cohen *d*=0.6) with existing eHealth interventions designed to address anxiety in children without long-term physical conditions such as BRAVE online (Cohen *d*=0.76) [28]. More than 85% (21/24) of our sample demonstrated clinical improvement immediately following intervention, and most maintained this benefit at the 3-month follow-up. *Starship Rescue* also appears to be more effective than Web-MAP, the only CBT-based, chronic pain–focused, web-based intervention identified by a recent Cochrane review for treating anxiety in children aged 11-17 years with long-term physical conditions (Cohen *d*=0.53) [67].

We believe that positive design features of *Starship Rescue* include a smaller number of modules (n=5) than other eHealth interventions for anxiety such as BRAVE online (n=16) and Cool Kids online (n=8), the reduction and tunneling of CBT content to improve adolescent engagement [68], and the inbuilt ability to repeat key skills to achieve mastery [69]. The lack of therapist support makes *Starship Rescue* more cost-effective than existing eHealth interventions for childhood anxiety. Despite the concern of other researchers that adherence may be diminished by the absence of clinician support [68], we did not find this to be the case in the context of this small trial. Objectively collected adherence rates are higher than those in other comparable eHealth interventions [70]. As we did not collect any parents’ feedback, we are uncertain whether their involvement optimized participant engagement and completion. We plan to explore this in an upcoming RCT. The study participants reported that they enjoyed biofeedback-based relaxation strategies. However, the potential additional benefit of combining biofeedback and CBT also remains unclear from these findings. Following more definitive evidence of its effectiveness, a head-to-head comparison of *Starship Rescue* and solely CBT and biofeedback interventions would be worthwhile to address this issue.

Despite being co-designed with its target audience, further minor modifications are warranted before the RCT to address some of the intervention’s technical aspects and improve its usability and acceptability. The fact that the mean length of time taken to complete the intervention exceeded expectations may be related to a number of issues. Extensive completion times for module 1 are likely to be because of participants being assisted by us to access module 1 during the onboarding process but not actually commencing or completing it until a later date. The delay in completing module 3 is likely to be related to participants getting stuck while playing the embedded platform game. Finally, although the requirement for real-world mastery of a chosen source of anxiety during module 4 may have proved challenging for users, this remains a vital means of generalizing therapeutic knowledge into practice [71]. Other key information from this pilot trial that will influence the subsequent RCT is the slow pace of recruitment from a single site by busy clinicians. For the RCT, recruitment from multiple sites is planned to ensure more timely data collection. The outcome measures used in this open trial appear suitable for use during RCT. Adherence to the intervention (defined by us as completion of all modules) was achieved by 67% (16/24) of the participants. This is comparable with previous studies of eHealth interventions, such as Smart, Positive, Active, Realistic, X-Factor Thoughts (60%), Cool Kids online (75%), and BRAVE online (85%), and a recent systematic review that identified a mean rate of completion of 64% for technologically delivered interventions for childhood anxiety and depression [42]. This is also encouraging, given the known association between module completion and outcomes for psychological eHealth interventions [72]. Although its reach may currently be limited by reliance on a physically worn heart rate monitor, emerging technology will likely permit biofeedback to be conducted via heart rate monitoring apps in the future.

### Strengths and Limitations

The strengths of this study are the co-design of *Starship Rescue* with end users, the inclusion of participants with different long-term physical conditions, and the small amount of missing data. The limitations of the study include the small sample size; the study being conducted in a single location, which may affect the generalizability of results; and the use of only self-reported outcomes. Exploration of parent or clinician ratings would also be valuable for comparison with user-rated levels of anxiety, as would an exploration of the types of anxiety participants choose to address; more detailed exploration regarding the combination of biofeedback and CBT; and differences in completion and acceptability between users of different genders and ethnicities during an adequately powered RCT. Given the difference between intervention use between experimental and nonexperimental settings [73], investigation of the intervention’s use in a naturalistic setting would be useful. Future research would benefit from formal economic analysis to bridge the gap between researchers’ interests and policy makers [74].

### Conclusions

*Starship Rescue* remains the only eHealth intervention specifically designed for treating anxiety and is evaluated with children and young people with long-term physical conditions. If future RCT results confirm the encouraging results from this pilot study, *Starship Rescue* has the potential to improve the short-term psychosocial well-being of this population by reducing psychological distress, improving quality of life, more optimal physical health management, reduced school absence, and improved social integration. In the longer term, it may also improve the rates of completed education, employment, and survival.

## Appendix

Multimedia Appendix 1 Description of content of the game and purpose of each module.

Multimedia Appendix 2 Change in symptom severity on the Generalized Anxiety Disorder Scale.

## Abbreviations

BRAVE: Body Signs, Relaxation, Active Helpful Thoughts, Victory Over Your Fears, Enjoy
CBT: cognitive behavioral therapy
EMA: ecological momentary assessment
GAD-7: Generalized Anxiety Disorder, 7-item
HRV: Heart Rate Variability
PedsQL: Pediatric Quality of Life Inventory
RCT: randomized controlled trial
SCAS: Spence Children’s Anxiety Scale
SUS: System Usability Scale
