# Supplementary material for: A Cognitive Behavioral Therapy–, Biofeedback-, and Game-Based eHealth Intervention to Treat Anxiety in Children and Young People With Long-Term Physical Conditions (Starship Rescue): Co-design and Open Trial
Source: JMIR Serious Games. 2021 Sep 24;9(3):e26084. doi: 10.2196/26084 (PMC8501411; doi:10.2196/26084)
Supplement: Multimedia Appendix 1 [file games_v9i3e26084_app1.docx]

**Supplementary File 1**

**DESCRIPTION OF CONTENT OF THE GAME AND PURPOSE OF EACH MODULE**

Table 1: Description of the content and core skills covered in each module (level) of Starship Rescue

| **Module** | **Content** | **Purpose** |
| --- | --- | --- |
| Module 1 | - Introduction to game, choice of avatar, choice of out-of-game reward - Mini-game 1: Finding the anxiety monster in the sea of emotions - Cavemen cartoon story - Mini-game 2: Exploration of the anxiety monster - Weapons chamber | Orientation; Understanding anxiety as one of a range of emotions; Understanding the origins of anxiety and symptoms of anxiety, including in oneself; Introduction to a range of coping mechanisms, including those that are already in use (strengths-based approach). |
| Module 2 | - Quiz to recap learning from module 1 - Hoverboard analogy - Introduction to biofeedback using wrist-band - Breath of power technique - Mini-game 3: Calm quest - Zero gravity technique - My happy place technique | Introduction to the 5-part model of CBT; Learning to use the body to beat anxiety via deep breathing, progressive muscle relaxation, and guided imagery; Practicing staying calm while playing a game. |
| Module 3 | - Quiz to recap learning from module 2 - Introduction to helpful and unhelpful thoughts - Astronaut cartoon story - Mini-game 4: Planet of the mind - Space detective analogy - Return to weapons chamber to practice breath of power, zero gravity, and/or my happy place techniques | Introduction to cognitive restructuring; Use of bicentric framework to learn from others; Encouragement to identify and rate thoughts as helpful or unhelpful; Reinforcement of concepts via a game. |
| Module 4 | - Quiz to recap learning from module 3 - Mini-game 5: Teleporter - Introduction to problem-solving - Mini-game 6: Space elevator - Introduction to graded exposure - Return to weapons chamber to practice breath of power, zero gravity and/or my happy place techniques | Introduction to problem-solving for simpler worries and graded exposure for more longstanding or difficult ones. |
| Module 5 | - Final quiz to place bravery stars into bridge of Starship and restart its engines - Emailing of Stay Cool Capsule with summary of learning | Consolidation of learning; Completion of game; Receipt of memory aid for use following the game. |

**THEORETICAL UNDERPINNINGS OF THE GAME**

Starship Rescue is underpinned by principles of (i) *CBT*, (ii) *biofeedback*, (iii) *learning theory*, and (iv) *game player taxonomy*.

1. *Cognitive Behavior Therapy* (CBT) involves recognizing the emotions, thoughts and bodily sensations associated with anxiety and learning to manage them using coping skills such as relaxation, thought restructuring, and exposure to triggers [43]. The five modules of Starship Rescue involve users i) learning how to identify and rate emotions, including anxiety; ii) learning how to use their body to beat anxiety; iii) learning how to use their minds to beat anxiety; iv) using their actions to beat anxiety and v) completing a final quiz to revise their knowledge about anxiety and its management.
2. (ii) *Biofeedback* utilizes electrical feedback on physiological processes and results in a change in bodily functioning. The precise mechanism of biofeedback is dependent upon the chosen modality. Heart rate variability (HRV), the interval between heartbeats, is determined by heart-brain interactions and non-linear autonomic nervous system (ANS) processes [44, 45]. The ANS consists of two parts, the sympathetic nervous system (SNS) and the parasympathetic system (PNS). During periods of anxiety, the SNS becomes activated, adrenaline is secreted into the bloodstream, and heart rate variability alters. These effects can be countered by regulating one's breathing and activating the PNS. Visual feedback of one's heart rate can make this process more concrete and accessible, even to children [46]. As there is a wide range of normal heart rates and HRV in people of different ages, during the development of Starship Rescue, it was deemed impossible to undertake sophisticated measurement of HRV. Instead, a recording of a player's heart rate when the wrist-band was worn in the first module became the baseline from which subsequent alterations were recorded when users played biofeedback-based games in subsequent modules.
3. (iii) *Learning theory* involves understanding how knowledge is acquired and utilized. Of the multiple learning theories in existence, Starship Rescue primarily draws on two: behaviorism and cognitivism. Behaviorism suggests that learning is based on external stimuli and subsequent responses [47]. Multiple aspects of the game engage the user in this manner, such as using reward-based games and regular quizzes. Cognitivism regards learning as an internal and active mental process that develops within a learner and increases mental capacity, skills, and further learning [48]. During Starship Rescue, users learn about the connection between thoughts and emotions, coping skills, and practice newly learned skills. In addition to these foundations, two other learning approaches were intentionally employed during the game's design. The first was the use of a bi-centric frame of reference. Demonstrated to be valuable for enhancing acquisition of abstract and multi-dimensional information, this combines the egocentric perspective, in which a user becomes immersed in an action (learning by doing) and the exocentric perspective in which they gain insights by distancing themselves from the context (being able to see the forest, rather than the trees) [49]. During Starship Rescue, users switch from completing tasks themselves (including games) to watching cartoons in which other characters experience and overcome similar worries. The final learning approach is situated learning in which authentic contexts, activities, assessment, and guidance are used to achieve 'transfer' of knowledge from one situation into another. This is the acid test of classroom-based learning [50] and has also been shown to be viable through well-designed digital contexts [51]. In the fourth module of Starship Rescue, users must apply learned anxiety-management skills to a real-life worry, and only when they complete this task, can they finish the game.
4. *Game player taxonomy* was originally described by Richard Bartle in the form of four key types of computer game players: Achievers, whose aim is to succeed (e.g., reach the highest level); Explorers, who enjoy interacting with the virtual world (e.g., finding and collecting objects); Socializers, who like interacting with other players/characters; and Killers, who like acting on other players/characters to gain either a good or bad reputation [52]. Starship Rescue incorporates games in which users must find an anxiety monster in the sea of emotions, kill guards along the way, collect crystals that contain helpful thoughts, and help the crew of a stranded spaceship restart its engines. A more recent user segmentation taxonomy proposed by Fleming et al. [53] is primarily related to age and spectrum of symptom severity; it divides potential health intervention users into gamers, engagers, skeptics, and straight talkers and recommends targeting game design to more specific audiences. During the co-design process for Starship Rescue, younger participants were noted to prefer game-based approaches. In contrast, older adolescents preferred more direct methods of learning in keeping with Fleming's observations. Given the median age of onset for anxiety disorders is 11 years [54], a decision was made to follow a game-based approach and target Starship Rescue to children aged 8-15. Due to ethics committee restrictions, the game's open trial was conducted with children aged 12-18 years, extended after a slow recruitment period to 10-18 years.
