# Supplementary material for: A Cognitive Behavioral Therapy–, Biofeedback-, and Game-Based eHealth Intervention to Treat Anxiety in Children and Young People With Long-Term Physical Conditions (Starship Rescue): Co-design and Open Trial
Source: JMIR Serious Games. 2021 Sep 24;9(3):e26084. doi: 10.2196/26084 (PMC8501411; doi:10.2196/26084)
Supplement: Multimedia Appendix 2 [file games_v9i3e26084_app2.docx]

Table S1: Change in symptom severity on GAD scale

|  |  | **Post** | | | | |
| --- | --- | --- | --- | --- | --- | --- |
|  |  | Subthreshold | Mild | Moderate | Severe | Total |
| **Pre** | Subthreshold | 1 | 0 | 0 | 0 | 1 |
|  | Mild | 5 | 2 | 0 | 0 | 7 |
|  | Moderate | 1 | 6 | 0 | 0 | 7 |
|  | Severe | 2 | 1 | 3 | 0 | 6 |
|  | Total | 9 | 9 | 3 | 0 | 21 |
|  |  | **3-month** | | | | |
|  |  | Subthreshold | Mild | Moderate | Severe | Total |
| **Pre** | Subthreshold | 1 |  |  |  | 1 |
|  | Mild | 5 | 3 |  |  | 8 |
|  | Moderate | 2 | 3 | 1 |  | 6 |
|  | Severe | 1 | 2 | 2 | 1 | 6 |
|  | Total | 9 | 8 | 3 | 1 | 21 |
